# Commentary: Molecular Machines Open Cell Membranes

**DOI:** 10.3389/fonc.2017.00277

**Published:** 2017-11-21

**Authors:** Shankargouda Patil, A. Thirumal Raj

**Affiliations:** ^1^Maxillofacial Surgery and Diagnostic Science, Division of Oral Pathology, College of Dentistry, Jazan University, Jizan, Saudi Arabia; ^2^Oral Pathology and Microbiology, Sri Venkateswara Dental College, Chennai, Tamil Nadu, India

**Keywords:** molecular targeted therapy, in vitro technique, cell membrane, lipid bilayer, ultra violet rays

The past decade has seen a substantial increase in the use of molecular targeted therapies. The major factor propelling research investments in molecular therapies is its ability to target specific cells with minimal damage to the surrounding tissues. The molecules used in targeted therapies have different functional roles ranging from delivering a therapeutic agent to causing cell death of the target cells through apoptosis or necrosis (1).

The first step in molecular therapy is for the molecule to accurately identify the target cells. The second step is to successfully deliver the intended effect on to the target cell. Although molecules currently in use have shown to have relatively high specificity in identifying the target cells, complications arise during the delivery stage, where in either the therapeutic agents carried by the molecule gets deactivated or released early or get conglomerated with other molecules and is eliminated by the host’s macrophages (2). Thus, to combat the disadvantages of the conventional targeted delivery systems, physical energy methods are being explored as possible means for effectively delivering the intended effect onto the target cells (2).

In molecular oncology, multiple techniques for the penetration of the cancer cell membrane is being explored to serve as tools for delivering the therapeutic agents. There is wide variation in the strategies used for the penetration of the lipid bilayers of the cellular membrane of the target cells. These include electric, magnetic, temperature, ultrasound, and light-based molecule activation systems (3–6). More recently, García-López et al. used molecules which on activation by external stimuli can produce mechanical actions with the capability to penetrate the bi-layered cell membrane of the target cell (1). The molecule consists of several components. The major portion of the molecule is the stator portion. Attached to the stator portion is the smaller rotor portion, which is activated by the UV light. Fluorophores are attached to the stator portion of the molecule to confirm the attachment of the molecule to the target cell. The molecules also carry short peptide addends to specifically identify the target cell through specific cell-surface recognition sites. The first step in the process of nanomechanics mediated targeted therapy is the adsorption of the action molecule on to the target cells lipid bilayer. The second step involves the UV based activation of the molecules. On exposure to 355–365 nm light, the rotor portion of the molecule gets activated and rotates at high frequency. Molecules without the rotor component are used as control (1). Due to the relatively smaller size of the rotors (1 nm) compared with the thickness of the bi-lipid membranes (7.5–10 nm), there is a delay period between the UV based activation of the molecule and the penetration of the target cell. This delay is seen even in other forms of probe-induced mechanical perturbations (7).

In most of the light absorbing molecules used in molecular therapies, following activation, the molecule merely transfers the absorbed light energy and dissipates it onto the target cell, which may not be sufficient for the desired disruption of the latter. The advantage in UV based nanomechanical force is that even if the target cell membrane is relatively more resistant to rupture, the force generated from the resulting motion of the molecular rotor is theoretically sufficient to disrupt the integrity of the cell membrane (1).

To conclude, García-López et al. have used *in vitro* techniques to successfully demonstrate the nanomechanical penetrating of cancer cells. Further animal based *in vivo* studies are required to substantiate the results of García-López et al. and to provide sufficient evidence to extend its application on to human trials.

## Author Contributions

AR drafted the manuscript. SP reviewed and edited the manuscript.

## Conflict of Interest Statement

The authors declare that the research was conducted in the absence of any commercial or financial relationships that could be construed as a potential conflict of interest.
